# Paranoid beliefs and conspiracy mentality are associated with different forms of mistrust: A three-nation study

**DOI:** 10.3389/fpsyg.2022.1023366

**Published:** 2022-10-18

**Authors:** Anton P. Martinez, Mark Shevlin, Carmen Valiente, Philip Hyland, Richard P. Bentall

**Affiliations:** ^1^Department of Psychology, The University of Sheffield, Sheffield, United Kingdom; ^2^School of Psychology, Ulster University, Coleraine, United Kingdom; ^3^Department of Personality Assessment and Clinical Psychology, Complutense University of Madrid, Madrid, Spain; ^4^Department of Psychology, Maynooth University, Maynooth, Ireland

**Keywords:** paranoid beliefs, conspiracy mentality, institutional trust, trustworthiness, interpersonal trust, trust in sources, multi-nation study

## Abstract

Paranoia and conspiracy are terms typically used interchangeably. However, although the underlying content of these types of beliefs might be similar (e.g., seeing others as powerful and threatening), recent research suggests that these constructs differ in important ways. One important feature shared by both constructs is excessive mistrust but this aspect might play different roles in each belief system. In this study we explored the strength of associations of different trust predictors (i.e., trust in institutions, trust in sources of information, perceptual trust, and interpersonal trust) between conspiracy mentality and paranoid beliefs. We tested this association in a large representative multinational sample (United Kingdom *n* = 2025; Spain *n* = 1951; and Ireland *n* = 1041). Confirmatory factor analysis supported a two-factor model of conspiracy and paranoid beliefs in each nation sample. Path and equality of constraints analysis revealed that paranoia was more strongly associated with perceptual mistrust (bias towards mistrusting unfamiliar faces) whereas conspiracy was more strongly associated with mistrust in political institutions. Although interpersonal mistrust and trust in social sources of information were associated significantly with conspiracy their association with paranoid beliefs was stronger. These findings clarify the role of different trust processes in both belief systems. Limitations of this study are discussed.

## Introduction

The terms “conspiracy theorist” and “paranoid” are often used interchangeably when referring to people who are suspicious of other people’s intentions, doubt the veracity of historical events, or who think that important governmental decisions are part of secret plots orchestrated by powerful others. For example, American historian Richard Hofstadter described the “paranoid style” of American politics as a sense of heated exaggeration, suspiciousness and conspiratorial fantasy, although emphasizing that he was not using the term “paranoid” in a clinical sense (Hofstadter, 1964). Some clinical definitions also appear to conflate the two concepts. For example, in the ICD-10 Classification of Mental and Behavioral Disorders, one of the characteristics of paranoid personality disorder was preoccupation with unsubstantiated “conspiratorial” explanations of events both immediate to the patient and in the world at large (World Health Organisation, 1993), although the concept was dropped from the later, 11^th^ edition (World Health Organization, 2018). However, most definitions of paranoia and conspiracy theories point to different conceptualizations of these constructs. Whereas the former refers to unfounded beliefs that involve intentional harm to the self from others (Freeman and Garety, 2000; Bentall et al., 2001), the latter is usually defined as an explanation for significant social and political events that involves secret plots by powerful and malevolent others (Douglas et al., 2017). Thus, although both constructs attribute events to the presence of threatening agents, and while there is consistent evidence that the two belief systems are modestly correlated (Imhoff and Lamberty, 2018; Alsuhibani et al., 2022), the locus of vulnerability for each appears to be different (i.e., the individual in the case of paranoia and society in general in the case of conspiracy theories; Greenburgh and Raihani, 2022; Greenburgh et al., 2022). It is estimated that nearly a third (26.7%) of the general population are convinced that there is a conspiracy behind many world events (Freeman and Bentall, 2017). It has also been observed that believing in a specific conspiracy theory is often associated with belief in many others (Goertzel, 1994; Swami et al., 2011), suggesting that conspiracy ideation can be considered a trait-like predisposition which is sometimes referred to as conspiracy mentality (Bruder et al., 2013). On the other hand, paranoid beliefs are not exclusive to clinical populations as they are experienced by at least 10–15% of the general population (Freeman, 2007) suggesting that they lie on a continuum from less severe to more dysfunctional forms (Bebbington et al., 2013; Elahi et al., 2017). Hence, researchers have tried to explore the psychological precursors of both types of beliefs by conducting studies in non-clinical populations. Factors such as negative self-esteem, disrupted attachment experiences, as well as various cognitive biases (i.e., jumping to conclusions or external locus of control) have been associated with the development and maintenance of paranoia (Bentall et al., 2014). Equally, narcissism, exaggerated positive view of the self, specific cognitive biases (i.e., confirmatory bias or illusory correlations), and poorer analytical reasoning have all been associated with conspiracy mentality (Douglas et al., 2017; Goreis and Voracek, 2019). Although conspiracy mentality and paranoid beliefs therefore appear to be associated with different specific psychological factors, both constructs are thought to involve excessive mistrust (Freeman and Bentall, 2017). Trust is considered to be a fundamental aspect of everyday social interactions (Simpson, 2007). By accepting being vulnerable to the actions of another party we hold positive expectations regarding the other party’s intentions and behaviors despite the uncertainty of what the outcome of that dyadic relationship will be (Lewicki et al., 2006; Lewicki and Brinsfield, 2012).These expectations are not restricted to interpersonal interactions but can extend to various social systems and large institutions (e.g., companies, banks, governmental agencies), or any circumstances in which we perceive another party as having control over our resources and life options (Simpson, 2007; Hatzakis, 2009). Thus, trust appears to be a key component in the way we build our social relationships as well as in social behaviors we engage in, ranging from voting for a political party, reading a specific newspaper or getting vaccinated.

The association between mistrust and paranoia is well established in the literature, the former being a subcomponent of the paranoia spectrum usually present in non-clinical populations (Bebbington et al., 2013; Bell and O’Driscoll, 2018). However theorizing and research about mistrust in paranoia usually focuses on interpersonal mistrust (Wickham et al., 2014; Furnham and Crump, 2015; Barreto Carvalho et al., 2017) and by extension to the untrustworthiness of unfamiliar faces (Kirk et al., 2013; Abbott et al., 2018; Martinez et al., 2021). On the other hand, conspiracy beliefs are usually linked to mistrust relating to society at large (Van Prooijen et al., 2021). For example, several studies have reported an association between conspiracy mentality and institutional mistrust, particularly in respect to political institutions (Kim and Kim, 2021; Mari et al., 2021). Nonetheless, a relationship between conspiracy mentality and lack of trust in other people has also been reported (Goertzel, 1994; Abalakina-Paap et al., 1999). One recent study reported that interpersonal mistrust was associated with both conspiracy mentality and paranoia, with the association between interpersonal mistrust and paranoia being stronger (Imhoff and Lamberty, 2018). This distinction between interpersonal and institutional mistrust is in line with the view that paranoia reflects a threat to the “self” whereas, in the case of conspiracy mentality, the threat is orientated to society more generally (Imhoff and Lamberty, 2018; Van Prooijen et al., 2021).

To the best of our knowledge, no study to date has explored the role of specific trust processes (i.e., interpersonal, institutional, trust in sources of information, trust perceptions) in relation to both conspiracy and paranoid beliefs using large representative samples. The current study aimed to expand our understanding of the relationships between conspiracy mentality and paranoid beliefs by examining how these beliefs co-varied with specific types of mistrust in representative population samples from three-nations: the United Kingdom, Ireland, and Spain. The data was collected as part of a multinational study of the psychological impact of the COVID pandemic, during the earliest stages of the emergency. Following the work of Imhoff and Lamberty (2018) and (Alsuhibani et al. 2022), we expected that paranoid beliefs and conspiracy mentality would form two distinct but correlated factors in all three-nation samples. We employed measures of a wide range of forms of trust (i.e., interpersonal, institutional, trust in sources of information, perceptual trust) and looked at the specific contributions of each to paranoia and conspiracy mentality. Thus, we hypothesized that conspiracy mentality would be specifically associated with institutional mistrust, in particular with political institutions, and with mistrust in traditional media as well as with mistrust in institutional sources of information. On the other hand, following the findings of (Martinez et al. 2021), we expected paranoid beliefs to be specifically associated with a tendency to judge face stimuli as untrustworthy (i.e., bias or perceptual mistrust). With regards to interpersonal mistrust, we predicted that this form of trust would be associated with both conspiracy mentality and paranoid beliefs however, we expected a stronger association with the latter.^1^

## Materials and methods

### Participants/procedure

This study was based on data collected in the first wave of parallel surveys conducted in the United Kingdom (*n* = 2025), Ireland (*n* = 1041), and Spain (*n* = 1951) as part of the COVID-19 Psychological Research Consortium (C19PRC)^2^ designed to monitor multiple indicators of psychosocial health during the early stages of the COVID-19 pandemic. Adult participants aged 18 years and over were recruited by the survey company Qualtrics in the United Kingdom and Ireland, and by SONDEA in Spain. In each country quota sampling methods were used to reach a representative sample stratified by age, sex, household income, and geographical distribution within the countries (for more methodological information about the C19PRC please see McBride et al., 2021; Spikol et al., 2021 and visit^3^ for United Kingdom, Irish and Spanish surveys respectively). Participants responded to questionnaires and other measures presented on the Qualtrics survey platform and measures were comparable in all three countries by design. Information regarding age, sex, ethnicity, and dates of data collection for each country is presented in Table 1. Specific information about whether participants had received a mental health diagnosis was not collected in this study.

**Table 1:** Sample characteristics, mean (*M)* standard deviation *(SD)* and percentages of sex distribution and ethnicity.

Country	Age		Sex		Ethnicity	Data collection dates	Announcement of 1^st^ lockdown
	*M*	*SD*	M %	F %			
United Kingdom	45.44	15.90	48.2	51.8	85.5% white British	March 23^rd^ – March 28^th^ 2020	23^rd^ of March 2020
Ireland	44.97	15.76	48.2	51.8	74.8% white Irish	March 30^th^ – April 5^th^ 2020	27^th^ of March 2020
Spain	45.13	12.81	52.8	47.2	93% Spanish	April 8^th^ – April 10^th^ 2020	14^th^ of March 2020

Ethical approval was granted by The University of Sheffield (Ref: 033759), the Social Research Ethics Committee at Maynooth University (Ref: SRESC-2020-2,402,202), and The Complutense University of Madrid (Ref: 2019/20–034) for the United Kingdom, Irish and Spanish samples, respectively. All participants were presented with an information page which detailed the purpose of the study, and confidentiality of their data (under GDPR guidelines), and their right to withdraw at any time.

### Measurements

*The Revised Paranoia and Deservedness Scale* (PADS-R; Melo et al., 2009). Paranoid beliefs were assessed by rating the agreement with five items of the PADS-R persecutory subscale ranging from 1 (*strongly disagree*) to 5 (*strongly agree*). Items involved statements such as *“I’m often suspicious of other people’s intentions towards me*” and *“People will almost certainly lie to me.”* This scale has been previously validated in clinical and general population samples and its internal reliability in this study was very good across three countries (United Kingdom, *α* = 0.86; Spain, *α* = 0.84; Ireland, *α* = 0.83).

*The Conspiracy Mentality Scale* (CMS; Imhoff and Bruder, 2014) was used as a measurement of conspiracy mentality in which participants have to rate how likely based on their opinion each statement is true from 0% (*certainly not*) to 100% (*Certainly*). This scale included five statements such as *“Events which superficially seem to lack a connection are often the result of secret activities”* and *“Many important things happen in the world, which the public is never informed about.”* The internal reliability of this scale across the three countries was very good (United Kingdom, *α* = 0.85; Spain, *α* = 0.83; Ireland, *α* = 0.84).

*Institutional trust*. Participants had to indicate to which extent they trusted the following institutions (1) Parliament (United Kingdom), Dáil Éireann (Irish Parliament), Congreso de diputados (Spanish Congress); (2) The government; (3) The police (United Kingdom), An Garda Síochána (Irish Police), La policía (Spanish Police); (4) The legal system; (5) Political parties; (6) Scientists; (7) Doctors and other health professionals. Responses ranged from 1 (*do not trust at all*) to 5 (*completely trust*). Items 1, 2 and 5 were combined and used as a measure of *Trust in political institutions* (United Kingdom, *α* = 0.87; Ireland, *α* = 0.88; Spain *α* = 0.84) whereas items 3 and 4 were used as a measurement of *Trust in legal institutions* (United Kingdom, *α* = 0.82; Ireland, *α* = 0.78; Spain *α* = 0.68) and with items 6 and 7 used as an indicator of *Trust in scientific institutions* (United Kingdom, *α* = 0.82; Ireland, *α* = 0.78; Spain *α* = 0.68).

*Trust in sources of information*. As with *Institutional trust,* participants were requested to indicate how much they trusted information from each of the following sources: (1) Newspapers, (2) Television,; (3) Radio, (4) Internet websites, (5) Social media, (6) Doctors, (7) Other healthcare professionals, (8) Government agencies, and (9) Family or friends. Responses were recorded on a Likert scale ranging from 1 (*do not trust at all*) to 5 (*completely trust*). Items 1 to 3 were combined and used as a measurement of *Trust in traditional sources of information* (United Kingdom, *α* = 0.78; Ireland, *α* = 0.83; Spain *α* = 0.86) whereas items 4, 5 and 9 were used as an indicator of *Trust in informal sources of information* (United Kingdom, *α* = 0.68; Ireland, *α* = 0.66; Spain *α* = 0.65) and items 6 to 8 were used as *Trust in institutional sources of information* (United Kingdom, *α* = 0.85; Ireland, *α* = 0.81; Spain *α* = 0.69).

*Facial trust detection task* (FTDT; Oosterhof and Todorov, 2008). To measure perceptual trust, data-driven computer-generated face stimuli were obtained from the University of Chicago Perception and Judgement Lab database.^4^ These faces have been previously validated in terms of apparent trustworthiness ranging from 1 (not at all trustworthy) to 9 (extremely trustworthy). From this database 6 faces calibrated as more trustworthy (+ 3 and + 2 SD) and 6 calibrated as less trustworthy (−3 and −2 SD) were selected and presented in random order. Participants were asked to indicate if they trusted each face. Responses were recorded in a binary way (Yes/No) to allow us to calculate the signal detection outcomes of bias (tendency to judge a trustworthy face as untrustworthy or *vice-versa*) and sensitivity (perceiver’s accuracy in discriminating trustworthy faces from untrustworthy ones). Computation of signal detection outcomes was based on Stanislaw and Todorov (1999) calculations (equation 1 for sensitivity and 7 for bias).^5^ Further details of this test and method of scoring are available in (Martinez et al. 2021).

*General interpersonal trust*. Participants were asked to indicate how much they agreed with the following statement “*Generally speaking, would you say that most people can be trusted or that you need to be very careful in dealing with people*?” on a 5 point Likert scale ranging from 1 (*Need to be very careful)* to 5 (*Most people can be trusted*). This item has been adapted from the European Values Survey (Inglehart et al., 2000).

*Neighborhood trust*. Respondents were asked to rate how comfortable they were when taking the following actions (1) *“Asking a neighbour to keep a set of keys to your home for emergencies”* and (2) *“Asking a neighbour to collect a few shopping essentials for you, if you were ill and at home on your own.”* The sum of both ratings scored on a 4-point Likert scale (from 1 “*very uncomfortable*” to 4 *“very comfortable”*) was used as a measurement of neighborhood trust. Internal reliabilities were good across three countries (United Kingdom, *α* = 0.84; Ireland, *α* = 0.83; Spain *α* = 0.82) These questions were taken from the United Kingdom Community Life Survey (Harper and Kelly, 2003).

### Statistical analysis

Our analyses aimed to (1) test the latent structure of paranoia and conspiracy mentality, (2) determine if the latent structure is invariant across countries, (3) determine if there are significant cross-country differences in mean levels of paranoia and conspiracy mentality, and (4) estimate the associations between trust variables and paranoia and conspiracy mentality, testing which associations are specific to one of these belief systems or the other.

The latent structure of paranoia and conspiracy was tested using confirmatory factor analysis, conducted on each of the national samples (United Kingdom, Spain, and Ireland), to compare two alternative models: Model 1, in which all the paranoia and conspiracy items loaded on a single factor (testing if conspiracy and paranoia represent the same underlying construct) and Model 2 in which paranoia and conspiracy were treated as separate but correlated factors. Once the best fitting model was determined, the cross-country invariance of the latent structure was tested using a multi-group model. Three, increasingly restrictive, levels of measurement invariance was tested; configural invariance which tests for the same factor structure, metric invariance where the factor loadings were constrained to be equal across the countries, and scalar invariance that tests for the equality of intercepts. Configural and metric invariance was evidenced if the fit of the models were acceptable, and the difference in fit between them was negligible. A criterion of −0.01 change in CFI and changes in RMSEA of 0.015 and SRMR of 0.030 have been suggested as criteria to evaluate measurement invariance (Putnick and Bornstein, 2016). To test for scalar invariance (differences in intercepts) a Multiple Indicator – Multiple Cause (MIMIC) model was specified to test for Differential Item Functioning (DIF). Scalar invariance would be evidence by the degree of DIF. Hence, a MIMIC model was specified using dummy coded nation variables (with United Kingdom nation as reference) as predictors of the paranoia and conspiracy mentality latent variables to determine if there were significant country differences in paranoia and conspiracy mentality. Then, a baseline model was defined where each direct path between the observed items and dummy-coded country variables were constrained to zero. DIF is the presence of “significant” direct effects from the dummy coded variables to the observed items. As proposed by Kaplan (1989) a combination of modification indices (MI) and standardized expected parameter change (SEPCs), with values higher than 10 and greater than 0.20 respectively, were used to determine which direct effects should be freely estimated in the model. Thus, the path with the greatest MI/SEPC was freely estimated in the model and then the model was re-estimated until there were no MI and SEPC values higher than 10 and 0.20.

To estimate the associations between trust variables and paranoia and conspiracy, all of the trust variables were added to the free estimated model as predictors of the latent variables. Given our large sample size and to assist with the interpretation of practical significance, we reported semi-partial correlations (*sr*) as they reflect the specific effect of each predictor variable on the dependent variable (Abdi, 2007; Dudgeon, 2016). Cohen (1992) criteria was used to interpret the magnitude of the effect, with *sr* values of ≥ 0.50, ≥ 0.30, ≥ 0.10, ≤ 0.09, considered large, medium, small and trivial, respectively. To test for specificity, equality constraints were tested using Wald tests, to determine if regression coefficients were significantly different.

All analyses were carried out in R 4.0.4 using the *lavaan* package *cfa* function (Rosseel, 2012) for conducting confirmatory factor analyses and for assessing measurement invariance (configural and metric), the *modindices* function for assessing MI and SEPC values, the *sem* function for calculating the regression models and the *lavTestWald* function for calculating equality of constraints between regression coefficients. The *fastDummies* package *dummy cols* function (Kaplan, 2020) was used for coding nation as dummy variables whereas the *ppcor* package *spcor* function (Kim, 2015) was used for calculating *sr* between predictor and outcome variables.

We report seven goodness of fit indices: the chi-square test; the Comparative Fit Index (CFI; Bentler, 1990); Tucker–Lewis Index (TLI; Tucker and Lewis, 1973); Root Mean Square Error of Approximation (RMSEA; Mac Callum et al., 1996); Standardized Root Mean Squared Residual (SRMR; Hu and Bentler, 1999); the Bayesian Information Criterion (BIC; Schwarz, 1978) and Akaike Information Criterion (AIC; Akaike, 1987). Non-significant Chi-square values, CFI and TLI values above 0.90, RMSEA and SRMR values smaller than 0.08, and lower AIC and BIC values were considered indicators of good model fit.

## Results

Fit indices for the confirmatory factor analysis model are shown in Table 2, supporting a two-factor model over a one-factor model in each of the national samples. Nonetheless, while most of the fit indices are within the cut-off criteria, it should be noted that the RMSEA values vary between 0.08 and 0.10 indicating that model fit is neither good nor bad (Hu and Bentler, 1999); hence these values should be interpreted with caution. Measurement invariance results shown in Table 3 confirmed that the two-factor model was supported across the three-national samples.

**Table 2:** Model fit paranoia and conspiracy one and two factor model.

Samples	Model	*χ* ^2^	*df*	*p*	CFI	TLI	AIC	BIC	RMSEA	SRMR
United Kingdom	1	4807.312	35	<0.001	0.496	0.352	76292.781	76405.048	0.259	0.215
	2	751.458^*^	34	<0.001	0.924^*^	0.900^*^	72238.927^*^	72325.807^*^	0.102^*^	0.055^*^
Spanish	1	4603.076	35	<0.001	0.455	0.299	70563.720	70675.242	0.259	0.227
	2	705.211^*^	34	<0.001	0.920^*^	0.894^*^	66667.854^*^	66784.952^*^	0.101^*^	0.057^*^
Irish	1	3851.702	35	<0.001	0.504	0.362	36161.009	36258.493	0.236	0.196
	2	320.385^*^	34	<0.001	0.925^*^	0.900^*^	34558.715^*^	34661.073^*^	0.093^*^	0.050^*^

* Indicates better model fit.

**Table 3:** Fit of measurement invariance (configural and metric) of two factor model.

	*χ* ^2^	*df*	CFI	TLI	AIC	BIC	RMSEA	SRMR	Δ*χ*^2^	Δdf	ΔCFI	ΔRMSEA	ΔSRMR
Configural	1688.933^***^	102	0.923	0.898	167807	168409	0.100	0.050	-	-	-	-	-
Metric	1892.804^***^	118	0.914	0.902	167978.9	168477.3	0.098	0.058	203.87^***^	16	0.009	0.002	0.008

* *p* < 0.05;

** *p* < 0.01;

*** *p* < 0.001.

Standardized paths from dummy-coded country variables to paranoia revealed significant differences in the factor means for United Kingdom and Spain (*β* = −0.388, *p* < 0.001) and for United Kingdom and Ireland (*β* = −0.103, *p* = 0.01). Likewise, standardized regression coefficients between dummy-coded country variables and conspiracy mentality were significant for United Kingdom and Spain (*β* = 0.613, *p* < 0.001) and for United Kingdom and Ireland (*β* = 0.147, *p* < 0.001). Results shown in Supplementary Table S1 reflect mean latent variable differences between nations regarding conspiracy mentality (higher in participants from Spain in comparison to participants from United Kingdom and Ireland) and paranoia (higher in participants from the United Kingdom in comparison to participants from Spain and Ireland). These values are in line with previously reported research with the same measures (Melo et al., 2009; Bruder et al., 2013; Đorđević et al., 2021; Alsuhibani et al., 2022).

Results from DIF analysis revealed the greatest MI/SEPC values corresponded to the direct path between the dummy-coded variable representative of Ireland and the third item of the conspiracy mentality questionnaire (…*government agencies closely monitor all citizens*; MI = 428.063, SEPC = 0.234). The model was run again with this path freely estimated revealing that the path between the dummy-coded variable representative of Ireland and the fourth item of the conspiracy mentality questionnaire exhibited the largest MI/SEPC values (…*events which superficially seem to lack a connection are often the result of secret activities*, MI = 375.601, SEPC = 0.194). Once the model was run again with this path freely estimated there were no longer large MI/SEPC suggestive of adding new free parameters to the model. The two paths freely estimated in the final model were statistically significant (item 3, *β* = 0.1768, *p* < 0.001; item 4, *β* = 0.1197, *p* < 0.001) nonetheless R-squared difference before and after the inclusion of these freely estimated paths was small accounting for 6% of the variance for item 3 (from 0.429 to 0.487) and 1.3% for item 4 (0.716 to 0.729). Fit statistics for these DIF models are presented in Supplementary Table S2.

Bivariate and semi-partial correlation coefficients, standardized regression coefficients, and Wald test statistics are shown in Table 4.

**Table 4:** Bivariate correlations, semi-partial correlations, standardised regression coefficients and Wald tests of equality of constraints from multivariate regression model predicting paranoia and conspiracy beliefs for whole sample controlling for nation.

Predictor		Variables	Conspiracy			Paranoia			Wald			*r*	*sr*	*β*(se)	*r*	*sr*	*β*(se)	
Institutional trust	Political	−0.34^***^	**−0.19** ^***^	**−0.204 (0.010)** ^***^	−0.04^*^	0.06^***^	0.059 (004)^***^	154.564^***^
	Scientific	−0.007	0.07^***^	0.071 (0.016)^***^	−0.24^***^	−0.06^***^	−0.066 (0.007)^***^	29.704^***^
	Legal	−0.20^***^	−0.05^***^	−0.081 (0.015)^***^	−0.19^***^	−0.05^***^	−0.074(0.007)^***^	5.909^*^
Trust in sources	Media	−0.12^***^	−0.05^***^	−0.083 (0.014)^***^	−0.03^*^	−0.07^***^	−0.094 (0.006)^***^	4.544^*^
	Informal	0.13^***^	**0.11** ^***^	**0.196 (0.015)** ^***^	0.08^***^	**0.16** ^***^	**0.221 (0.007)** ^***^	25.587^***^
	Institutional	−0.09^***^	−0.05^***^	−0.094 (0.014)^***^	−0.16^***^	−0.08^***^	−0.104 (0.006)^***^	6.546^*^
Perceptual trust	Bias	−0.09^***^	−0.007	−0.025 (0.027)	−0.25^**^	**−0.12** ^***^	**−0.138 (0.012)** ^***^	5.712^*^
	Sensitivity	0.01	0.01	0.015 (0.022)	−0.004	0.01	0.015 (0.010)	0.475
Interpersonal trust	General	−0.19^***^	**−0.12** ^***^	**−0.160 (0.024)** ^***^	−0.35^***^	**−0.22** ^***^	**−0.272 (0.011)** ^***^	5.393^*^
	Neighborhood	−0.05^***^	0.05^***^	0.057 (0.012)^***^	−0.16^***^	−0.06^***^	−0.088 (0.006)^***^	41.7615^***^
Nation (United Kingdom)	D1 (Spain)	0.26^***^	**0.12** ^***^	**−0.164 (0.061)** ^***^	−0.13^***^	**−0.14** ^***^	**−0.227 (0.028)** ^***^	189.361^***^
	D2 (Ireland)	−0.07^***^	0.06^***^	−0.055 (0.067)^***^	0.04^**^	−0.01^*^	−0.040 (0.028)^**^	4.582^*^
*R^2^*		0.239			0.257			

Bolded values represent practical significant coefficients based on semi partial correlations (sr).

* *p* < 0.05;

** *p* < 0.01;

*** *p* < 0.001.

A significant, small to medium effect was found between mistrust in political institutions and conspiracy mentality (*sr* = −0.19) whereas the effect between mistrust in political institutions and paranoia, although significant, was very small (*sr* = −0.06). This difference was reflected in a significant Wald test showing that the association between mistrust in political institutions and conspiracy mentality was stronger than the association of the same predictor with paranoia. Examining the associations between the rest of the institutional trust predictors (scientific and legal) and paranoia and conspiracy mentality, we found significant but very small effects (*sr* < 0.08).

Regarding the associations between trust in sources of information and the two belief systems, significant but small positive effects were found between trust in informal sources of information and conspiracy mentality (*sr* = 0.11) and also paranoia (*sr* = 0.16). However, a significant Wald test revealed that the association between trust in informal sources of information and paranoia was stronger than the association between the same trust predictor and conspiracy mentality. On the other hand, the associations between trust in other sources of information (traditional media, institutional sources) and paranoia and conspiracy mentality, although significant, were very small (*sr* < 0.09).

In the case of perceptual trust, a significant relationship was found between a bias towards judging face stimuli as untrustworthy and paranoia (*sr* = −0.12), but not conspiracy mentality. However, no significant associations were found between both belief systems and the sensitivity of trust/mistrust judgments.

When considering interpersonal trust, a significant but small negative association was found with conspiracy mentality (*sr* = − 0.12) whereas a higher significant negative association was found with paranoia (*sr* = − 0.22), indicating low levels of interpersonal trust in relation to both types of beliefs; a significant Wald test indicated that the latter association was stronger than the former. Finally, regarding neighborhood trust, a significant positive association was found with conspiracy mentality (people higher in conspiracy mentality trusted their neighbors more) but a significant negative association with paranoia was also found. The Wald test revealed that this difference was significant but both effects were very small (*sr* = 0.05; *sr* = − 0.06, respectively).

## Discussion

In this study, we first examined if paranoid beliefs and conspiracy mentality were two separate but correlated phenomena and found that, as expected, a two-factor model was superior to a single-factor model in three large representative nation samples. These findings are in line with those of Imhoff and Lamberty (2018) and (Alsuhibani et al. 2022). However, whereas several studies have reported moderate to high associations between conspiracy mentality and paranoid ideation (Grzesiak-Feldman and Ejsmont, 2008; Darwin et al., 2011; Bruder et al., 2013; Barron et al., 2014; Brotherton and Eser, 2015; Cichocka et al., 2016) the correlation we observed (*r* = 0.11) between these constructs was much smaller. Thus, while the two-factor model provided optimal fit, the correlation between the factors strongly points towards two, distinct factors. In this context, we note that our sample was much larger, more international, and more representative of the participating nations than any hitherto study (most of the aforementioned studies used student or convenience samples). Moreover, some of these earlier studies measured paranoia within the context of schizotypal traits which are usually regarded as an expression of a latent psychopathological entity (e.g., schizophrenia spectrum disorder; American Psychiatric Association, 2013). Although the authors of these articles do not regard conspiracy theories as a reflection of an underlying psychopathology, they consider that certain psychopathological traits might facilitate the belief in conspiracy theories (Darwin et al., 2011; Swami et al., 2013; Barron et al., 2014). Contrary to this view, authors such as the philosopher Cassam (2019) argue that studying conspiracy theories from an individual differences perspective fails to address one of the most important features of these theories, which is that they are often politically motivated. From this perspective, conspiracy theories can be thought of as ideologies (i.e., set of ideas and beliefs) that structure the understanding of the political world, and thus considering them as a trait of an underlying psychopathology underestimates the social harm that can cause (Cassam, 2019). In future research, it would be useful to compare how paranoia and conspiracy theories relate to political psychology variables, for example authoritarianism, collective mistrust, mistrust to specific outgroups, mistrust to political figures and authorities, which we anticipate would be associated with the latter and not the former.

As a second aim, we explored the association between different types of trust variables and conspiracy mentality and paranoid beliefs. Our expectations regarding conspiracy mentality were partially met as we found an association between conspiracist thinking and mistrust in political institutions whereas associations with trust in scientific or legal institutions were trivial. Moreover, whereas conspiracy mentality was related to trust in informal sources of information (i.e., friends, social media) associations with trust in traditional and institutional sources of information were very weak. On the other hand, our expectations in relation to paranoia were supported by our findings, as higher paranoid beliefs were associated with a bias towards judging unfamiliar faces as untrustworthy. Furthermore, interpersonal mistrust was associated with both conspiracy and paranoia, with Wald tests revealing that this relationship was stronger for the latter. Finally, and surprisingly, we found that paranoia, more than having a conspiracy mentality, was associated with trust in informal sources of information.

Some of our findings appear to be in line with previous research whereas others seem less consistent, particularly in relation to conspiracy mentality. Authors such as Pierre (2020) have suggested that conspiracy theories are the consequence of epistemic mistrust, which is defined as mistrust of knowledge from authoritative sources (e.g., government). When disregarding information from well-established institutions, conspiracy theorists look for alternative explanations of events (Abalakina-Paap et al., 1999; Pierre, 2020; Meuer and Imhoff, 2021). Following this line of thought, authors such as Hartman et al. (2021) argue that circumstances related to global uncertainty, for example the COVID-19 pandemic, are particularly likely to give rise to conspiracy theories, although different specific conspiracy theories may be associated with different specific factors. For example, various studies have shown that distrust in governments is usually associated with general conspiracy theories (Goertzel, 1994; Pierre, 2020; Mari et al., 2021) while distrust in scientific or other institutions tends to be associated with specific conspiracies, for example about COVID-19 or HIV (Ball et al., 2013; De Coninck et al., 2021; Hartman et al., 2021). Our findings are consistent with this previous research, as mistrust in political institutions (i.e., government, political parties and parliament) was associated with conspiracy mentality but the relationship between conspiracist thinking and mistrust in scientific and legal institutions was trivial. Although some studies have found that conspiracy ideation is related with mistrust in traditional media (Freeman et al., 2020; van der Linden et al., 2020) others have reported no association at all (De Coninck et al., 2021). Our results are aligned with those of those of De Coninck et al. (2021) who found that conspiracy mentality was associated with relying on social media and personal contacts when gathering information about COVID-19.

Conspiracy theories are generally transmitted through social networks, online blogs and social media use (Parsons et al., 1999; Stempel et al., 2007; De Coninck et al., 2021; Enders et al., 2021; Mari et al., 2021) and it is therefore unsurprising that someone with a conspiracy mentality would be more likely to trust their own sources of information. However, paranoia often occurs in the context of loneliness (Alsuhibani et al., 2022), social isolation (Butter et al., 2017), and insecure attachment (Wickham et al., 2014) so a positive association with trust in informal sources of information is surprising in the case of this kind of belief. Possibly, people with high paranoia tend to trust their own personal sources of information given the potential anxiety elicited by face to face contact with others outside of family and friends circles. More studies are needed that includes measurements of social anxiety, social isolation, attachment and size of social networks in order to test these alternative explanations to our findings. In our study, the associations between both conspiracy mentality and paranoia and mistrust in official sources of information was very small. Previously, one multi-study article has reported moderate to strong correlations between paranoia and this kind of mistrust (e.g., government, mainstream media, scientists; van der Linden et al., 2020). The authors of this article argue that distrust in formal sources of information and paranoia are mechanism that explain the association between political conservatism and conspiracy thinking, supporting Hofstadter’s (1964) idea of the paranoid style in American politics (van der Linden et al., 2020). However, it has been previously argued that Hofstadter misunderstood the nature of paranoia, and that what he was really referring to was conspiracist thinking (Alsuhibani et al., 2022).

Interpersonal mistrust was also associated with conspiracy mentality but not as strongly as with paranoia, a finding which is aligned with previous studies of conspiracy theories (Goertzel, 1994; Abalakina-Paap et al., 1999; Green and Douglas, 2018; Meuer and Imhoff, 2021) and paranoia (Kramer, 1994; Axelrod et al., 1997; Murphy et al., 2012; Wickham et al., 2014; Furnham and Crump, 2015; Kong, 2017; Greenaway et al., 2019). By contrast, a bias towards judging unfamiliar faces as untrustworthy was specifically related to paranoid beliefs, replicating the finding of (Martinez et al. 2021). In dangerous and uncertain contexts, a tendency to classify social cues as untrustworthy would be an evolutionary adaptive strategy, as it would enable individuals to avoid the highly costly consequences of underestimating threat (Haselton and Buss, 2000; Haselton and Nettle, 2006). This bias is consistent with current models of paranoia, which assume that the expectation of harm from others is its core feature (Hooker et al., 2011; Kirk et al., 2013; Martinez et al., 2021). Consistent with this account, studies using game theory paradigms have found that paranoia is associated with an increased tendency to make attributions of harmful intent, leading researchers to argue that people with paranoid beliefs are prone to make these kinds of attributions when they experience marked uncertainty about the world (Greenburgh et al., 2019; Barnby et al., 2020). Conversely, a recent study by Meuer and Imhoff (2021) failed to provide evidence that conspiracy theories were related to the detection of social threat. Thus, our findings support the view that paranoia reflects self-relevant concerns as opposed to conspiracy theories which involves societal ones (Imhoff and Lamberty, 2018).

### Limitations

This study has some limitations. Firstly, our design is cross-sectional, which limits our ability to make causal claims. Future studies could benefit from employing experimental or longitudinal designs when studying causal relationships between trust processes and paranoid and conspiracy mentality. Moreover, the use of computer-generated face stimuli for measuring trustworthiness of unfamiliar faces might lack ecological validity and generalizability as the face stimuli were male, bald and Caucasian. This lack of ecological validity can also be applied to the self-report instruments used to measure interpersonal trust as they cannot tap into the social-interactive nature of interpersonal trust processes. Thus future studies would benefit from including socially interactive measures such as game theory paradigms or virtual reality scenarios. Finally, this data was collected during the very beginning of the first national lockdown during the first wave of the COVID-19 pandemic (Table 1). Since paranoid and conspiracy beliefs are thought to be heightened during threating and uncertain circumstances (Freeman, 2007; van Prooijen and Jostmann, 2013) it is possible that these beliefs were affected by the global impact the pandemic had at a societal and personal level.

We found that mean levels of conspiracy and paranoid beliefs differed significantly between the three-nation samples. Paranoid levels were higher in United Kingdom and Ireland than those reported in Spain. Conversely, the United Kingdom and Ireland reported lower levels of conspiracy endorsement than Spain (Supplementary Table S1). These differences might be due to various factors, such as degree of restriction enforcement by governmental institutions, perception of personal threat caused by the virus and habituation to the situation. The Spanish population had been quarantined for almost 3 weeks when the participants from that country completed the survey. Thus, it is possible that, in Spain at that time, the virus was not as threatening to individuals as COVID restrictions were, fueling feelings of discontent towards the government. In contrast, United Kingdom and Irish participants completed the survey shortly after lockdown was announced and thus the threat from the virus might have been associated with a high level of interpersonal vulnerability. Another interpretation of these findings could be in terms of cultural differences between individualistic (United Kingdom, Ireland) and collectivist (Spain) countries. Whereas the former might orientate external threats to the self the latter might focus those threats to society, resulting in higher conspiracy mentality in Spain and higher paranoid beliefs in Ireland and United Kingdom. However, this interpretation is highly speculative, further research would be required to test it, and future studies should therefore consider including instruments measuring cultural variables.

## Conclusion

This study employed large representative samples from three different countries allowing us to conduct high powered statistical tests as well as to generalize our results to the general population. Our findings show that paranoid beliefs and conspiracy mentality are two related but separated constructs and that this relationship between the two constructs did not differ between countries. Moreover, we found that conspiracy mentality and paranoid beliefs have shared and different trust predictors, with mistrust in political institutions being specifically associated with conspiracy mentality whereas a bias towards mistrust was uniquely associated with paranoid beliefs. Interpersonal mistrust and trust in informal sources of information were related with both paranoia and conspiracy mentality although the association of both trust predictors was stronger with paranoid beliefs. Our findings clarify the role of different trust mechanism in the two belief systems. Whereas conspiracy beliefs are conceptualized as ideologies which serves a political function as a response to social vulnerability (Abalakina-Paap et al., 1999; Miller et al., 2016; Cassam, 2019) paranoid beliefs reflects a cognitive structure that underlies personal vulnerability in which others are regarded as threatening. These findings may potentially lead to a better conceptualization of paranoia which will lead to more accurate assessment and more targeted interventions in clinical settings for example, by focusing specifically on interpersonal and perceptual mistrust. Further research is needed in order to replicate these findings and to establish the causal processes that are responsible for the associations reported here.

## Data availability statement

The datasets presented in this study can be found in online repositories. The names of the repository/repositories and accession number(s) can be found at: https://osf.io/sqgfw/.

## Author contributions

AM: conceptualization, formal analysis, methodology, and writing – original draft. MS: statistical conceptualization, supervision, writing, review and editing. CV and PH: writing, review, and editing. RB: conceptualization, methodology, writing – original draft, supervision, review, and editing. All authors contributed to the article and approved the submitted version.

## Funding

Data collected in the United Kingdom was supported by funding from The University of Sheffield, Ulster University and subsequently supported by the Economic and Social Research Council (grant ref. ES/V004379/1). Data collected in Ireland was funded by the Health Research Board and the Irish Research Council under the COVID-19 Pandemic Rapid Response Funding Call (COV19-2020-025). Data from Spain was supported by grants from the Ministry of Science and Innovation (PSI2016-74987-P) and Instituto de Salud Carlos III (COV20/00737) and funds from the UCM for consolidated research groups (GR29/20).

## Conflict of interest

The authors declare that the research was conducted in the absence of any commercial or financial relationships that could be construed as a potential conflict of interest.

## Publisher’s note

All claims expressed in this article are solely those of the authors and do not necessarily represent those of their affiliated organizations, or those of the publisher, the editors and the reviewers. Any product that may be evaluated in this article, or claim that may be made by its manufacturer, is not guaranteed or endorsed by the publisher.

## Supplementary material

The Supplementary material for this article can be found online at: https://www.frontiersin.org/articles/10.3389/fpsyg.2022.1023366/full#supplementary-material

## Footnotes

^1^Hypotheses of this study have not been pre-registered.

^2^ https://www.sheffield.ac.uk/psychology-consortium-covid19

^3^ https://osf.io/2y45r

^4^ https://tlab.uchicago.edu/

^5^For more information about signal detection calculations see: Stanislaw, H., & Todorov, N. (1999). Calculation of signal detection theory measures. Behavior research methods, instruments, & computers, 31(1), 137–149.
