# Supplementary material for: Paranoid beliefs and conspiracy mentality are associated with different forms of mistrust: A three-nation study
Source: Front Psychol. 2022 Oct 18;13:1023366. doi: 10.3389/fpsyg.2022.1023366 (PMC9623260; doi:10.3389/fpsyg.2022.1023366)
Supplement: Supplementary file 1 [file Table_1.docx]

| **Table S1.**  Mean differences between nations. Pairwise comparison with Bonferroni correction for paranoia and conspiracy. | | | | | | |
| --- | --- | --- | --- | --- | --- | --- |
| **Variables** | **Nation** | | | **Pairwise Comparisons** | **Mean difference**  **95%CI [L/U]** | ***p*** |
|  | *UK* | *Spain* | *Ireland* |  |  |  |
|  | Mean (SD) | Mean (SD) | Mean (SD) |  |  |  |
|  |  |  |  |  |  |  |
| Paranoia | 7.43 (5.77) | 5.96(4.43) | 7.26(4.87) | UK-Spain | -1.47 [-1.86/-1.07] | <.001 |
|  |  |  |  | UK-Ireland | -.017 [-.06/.03] | 1.00 |
|  |  |  |  | Ireland-Spain | 1.30 [.82/1.78] | <.001 |
|  |  |  |  |  |  |  |
|  |  |  |  |  |  |  |
|  |  |  |  |  |  |  |
| Conspiracy | 35.17 (9.17) | 40.69 (9.20) | 36.37 (9.26) | UK-Spain | 5.52[4.81/6.24] | <.001 |
|  |  |  |  | UK-Ireland | 1.20[.32/2.07] | <.001 |
|  |  |  |  | Ireland-Spain | -4.32[-5.19/-3.45] | <.001 |
|  |  |  |  |  |  |  |

**SUPPLEMENTARY MATERIAL**

Martinez et al., (2022)^[[1]](#footnote-1)^

| **Table S2.** Mean and standard deviations (SD) for the combined sample as well as same statistics reported in other studies | | | | | |
| --- | --- | --- | --- | --- | --- |
| **Variables** | *Combined Sample*  Mean (SD) | *Melo et al (2009)*  Mean (SD) | *Al-Suhibani et al (2022)*  Mean (SD) | *Bruder et al (2013)*  Mean (SD) | *Đorđević et al (2021)*  Mean (SD) |
|  |  |  |  |  |  |
| Paranoia | 1.37 (1.02) | 1.18 (.078) | 1.69 (0.95) | - | - |
|  |  |  |  |  |  |
| Conspiracy | 7.51 (1.90) | - | 6.91 (2.11) | 6.48 (2.40) | 7.88 (2.26) |
| *Note.* All values have been averaged based on the number of total items that the authors have used in order to make the results easier to interpret. | | | | | |
|  | | | | | |

| **Table S3.** Fit Statistics for MIMIC model | | | | | | | |
| --- | --- | --- | --- | --- | --- | --- | --- |
| **Model** | ***ꭓ^2^*** | **df** | ***p*** | **CFI** | **TLI** | **RMSEA** | **SRMR** |
| Baseline constrained | 2449.638 | 50 | <.001 | .898 | .867 | .098 | .057 |
| Ireland -> CMQ Item3 | 1990.389 | 49 | <.001 | .918 | .891 | .089 | .048 |
| Ireland -> CMQ Item4 | 1595.736 | 48 | <.001 | .934 | .911 | .080 | .042 |

1. doi: 10.3389/fpsyg.2022.1023366 [↑](#footnote-ref-1)
